# Electrical Sintering of Silver Nanoparticle Ink Studied by *In-Situ* TEM Probing

**DOI:** 10.1371/journal.pone.0017209

**Published:** 2011-02-24

**Authors:** Magnus Hummelgård, Renyun Zhang, Hans-Erik Nilsson, Håkan Olin

**Affiliations:** 1 Department of Natural Sciences, Engineering and Mathematics, Mid Sweden University, Sundsvall, Sweden; 2 Department of Information Technology and Media, Mid Sweden University, Sundsvall, Sweden; Queen's University at Kingston, Canada

## Abstract

Metallic nanoparticle inks are used for printed electronics, but to reach acceptable conductivity the structures need to be sintered, usually using a furnace. Recently, sintering by direct resistive heating has been demonstrated. For a microscopic understanding of this Joule heating sintering method, we studied the entire process in real time inside a transmission electron microscope equipped with a movable electrical probe. We found an onset of Joule heating induced sintering and coalescence of nanoparticles at power levels of 0.1–10 mW/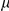
m^3^. In addition, a carbonization of the organic shells that stabilize the nanoparticles were found, with a conductivity of 4 10^5^ Sm^−1^.

## Introduction

Printed electronics is a low-cost alternative to standard silicon technology. Several applications are of interest, for example, as an electronic replacement to the simple barcode tags printed on products for identification. Price is the limiting factor and while a bar-code cost around 0.5 cent to print on a box, the electronic version, passive radio frequency identification (RFID) tags from the silicon industry, have now reached a lowest price of 5 cents, which is a too high price for most applications [1]. Printing with ink is a cheaper technology compared with silicon chip production and therefore the concept of printed electronics could serve as the needed low-cost alternative.

Printing by depositing molten metal droplets is problematic, mainly due to the technical difficulties at the high temperature required, leading to problems with adhesion or oxidation of the metal [2], [3].

Inkjet technology is therefore used in most approaches, mainly for printing electrically conductive pathways [4] but also for more advanced structures like thin film transistors [5]. Commercially available inkjet printers are used together with different types of inks, mainly based on nanoparticles. These nanoparticle inks consist of a solvent (organic or inorganic) together with metallic nanoparticles usually made out of silver [6]–[15], gold [15]–[21], and recently copper [22], [23].

The nanoparticles are typically in the range 1–100 nm. The smallest sizes, i.e. less than 10 nm, have the advantage of lower melting temperatures [24]. The solvent serves as a stabilizing matrix for the nanoparticles and forms protective shells around them to prevent them from coalescence. The solvent is also designed to satisfy the requirements of the printing nozzle such as drying and viscosity constrains.

Printing using nanoparticle inks results in a too low electrical conductivity of the printed structures, especially for low resistance demanding structures such as antennas. To directly melt the nanoparticles in order to increase the conductivity would demand a too high temperature (962°C for silver), for most printed substrates, such as paper, to survive. Therefore a sintering step is used to improve the conductivity, usually the sintering temperature is between 50% to 80% of the melting temperature of the material, but for nanosized powders the sintering temperature can be even lower, i.e. 20% to 30% of the melting temperature [25].

Usually a furnace is used for sintering, typically at 100–300°C. This heating step does also introduce substrate shrinkage and is time consuming. Control of the percolation threshold is important to prevent too much substrate shrinkage which can result in microcracking [26]. Visible-near IR spectroscopic ellipsometry has been suggested as one method to probe percolation transition during the sintering [27].

To replace the furnace, laser [16], [23], [28], microwave radiation [7], light flashing by utilizing the photothermal effect of nanostructures [29], [30], and chemical methods have been developed [9], [11], [14], [21].

A resistive heating process has also been demonstrated [26], [31] and in this Joule heating method, a voltage is applied to the printed structure, and the current heats the structure locally between the electrodes. The Joule heating method can be another way to control the percolation by tailoring the degree of sintering due to in-situ control of the heating current. This method can also be used to determine conducting pathways within a thin film using a movable electrode.

In this Joule heating method, much higher local power densities can be used compared to that in a furnace. The process may even pass the first coalescence stage, where the nanoparticles are just making contact, forcing the nanoparticles to sinter, leading to an increase in grain sizes [10], [31].

The Joule heating studies made so far have mainly used macroscopic methods and it is unknown how the process works at the nanoscale, for example, how individual nanoparticles coalescence or sinter and the corresponding change in electrical conductivity. It is also not clear what is happening to the dispersion matrix in the high power environment of Joule heating.

Here, we report on a microscopic study of electrical sintering of nanoparticle inks using in-situ transmission electron microscopy (TEM) probing. This instrument is essentially a small scanning tunneling microscope (STM) inside a TEM, where the TEM is used for imaging, while the movable STM tip provides a tool for electrical probing and manipulation [32]–[34]. This TEM-STM tool allowed us to study the sintering process of individual nanoparticles and we found an onset of sintering and coalescence at power densities of 0.1–10 mW/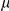
m^3^. Furthermore, we found a carbonization of the protective shells covering the nanoparticles that were particularly visible when the metal nanoparticles were completely evaporated. This carbon structure had a conductivity similar to graphite.

## Methods

We used a silver ink, Silverjet DGP-40LT-15C, with particles size of 59±2 nm (Advanced Nano Products). The dispersion matrix of the ink is a polar solvent (triethylene glycol monoethyl ether).

Gold wires of 0.25 mm in diameter, were cut into sharp tips by cutting at a 45° angle with a pair of scissors. We applied the ink onto the gold wires by dipping the wire into the ink, let to dry, and thereafter placed into an in-situ TEM side entry holder [35] (Nanofactory Instruments). This special side entry holder utilizes two electrodes, one that is static and one that is movable. A gold-wire with the dried ink serves as one of the electrodes (static) while the other electrode (movable) is a clean gold wire without ink. The TEM used was a JEOL-2000FX at 160 kV with digital camera that allowed for movie recording during the experiments.

**Figure 1:**
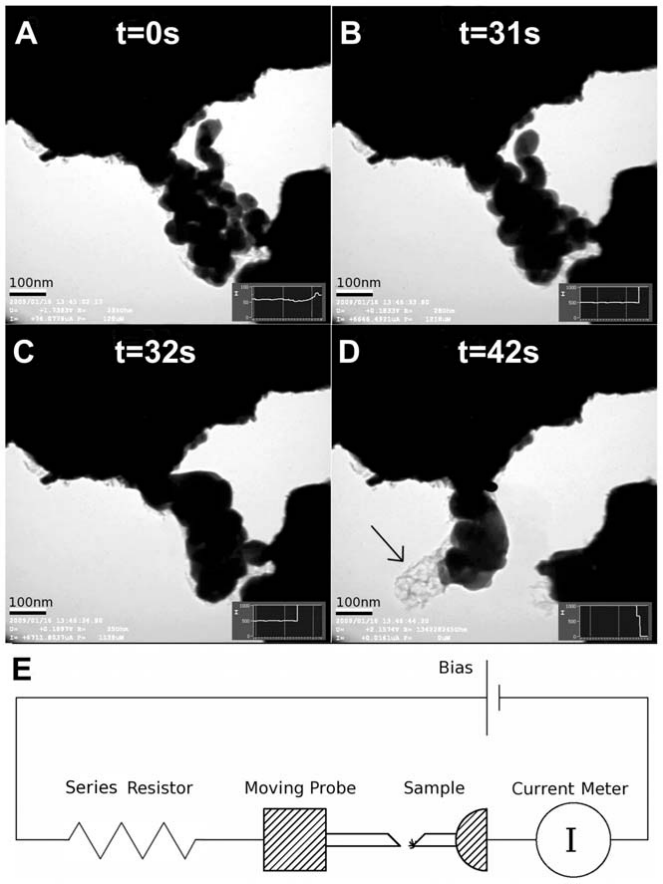
TEM images of the process of electrical sintering and coalescence of silver nanoparticle ink. During increasing electrical current: a) Starting sample with a initial resistance 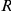 of 200 k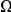 that slowly drops to 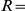
15 k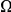. b) Sintering of nanoparticles, that occurred in less than one seccond. 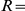
30 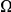 c) Grain growth of nanoparticles, 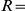
20 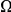 d) Evaporation of silver. The arrow points to a carbonized net-like structure. e) Schematic drawing of the in-situ TEM probe electrical wiring configuration.

**Figure 2:**
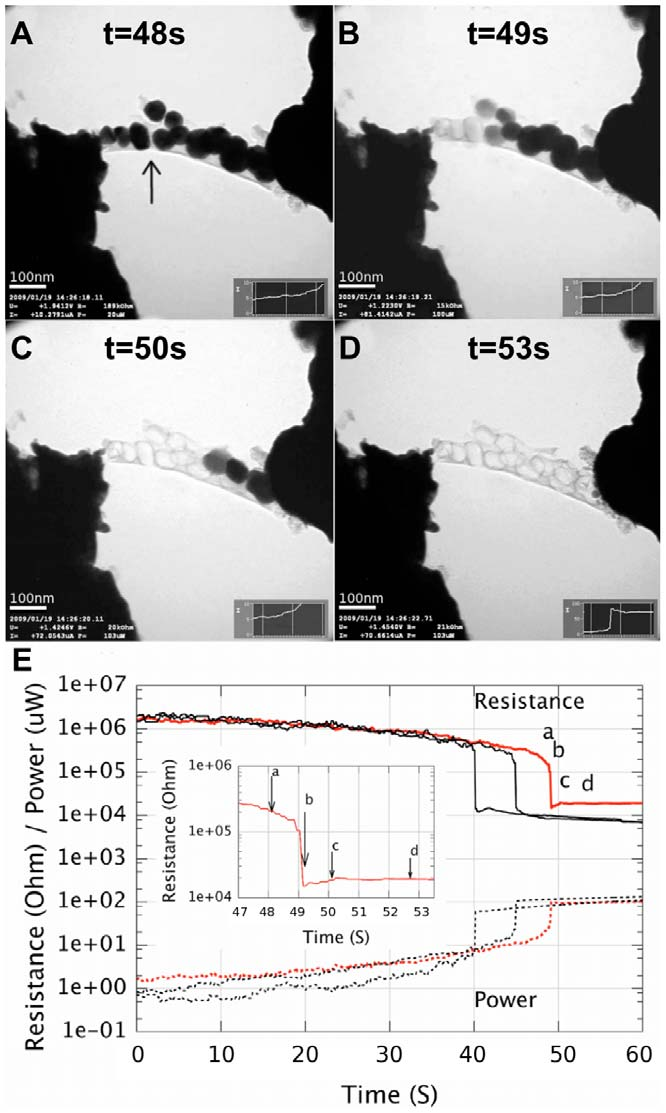
TEM-images showing the carbonization and sintering process correlated to measured electrical data. a) before sintering and b–d) after sintering when the particles melts way in which the remaining carbonized net-like structure is easily seen. The scale bar in all images is 100 nm. e) The corresponding resistance and Joule power as a function of time for three different samples. The lettered curve and the inset curve, correspond to the TEM image sequence.

The samples had either the nanoparticles arranged in a clusters form (figure 1) or like a necklace (figure 2). These configuration of nanoparticles was self-formed during the ink dipping and drying procedure and on the surface of the ink coated gold wire, severals of these obstacles can be seen pointing outwards when viewing in TEM. By using the other movable electrode, during TEM-session, electrical contact to single obstacles were establish. This configuration with the sample bridging between the two electrodes gives an electrical two probe setup (figure 1e).

A voltage, 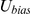, was applied to the sample and was part of an electrical circuit together with a series resistor 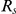 (300 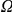 or 10 k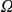) which limited the current, 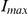, through the sample (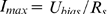). This to prevent the positive feedback that occurs when resistance drops in the sample due to the sintering that otherwise would have led to an increased power input and failure [36].

The bias voltage was slowly increased, up to 10 V, while measuring the current that were recorded together with movies (10 frames/s) of the TEM-images. The movie recording, electrical data measurement, and control of the probe were all done using a LabView program together with a PXI-measurement system.

Detailed studies were done on seven samples with lengths of 200–600 nm. Four of the experiments were of cluster type and the other three of necklace configuration. For each experiment a new ink-dipped gold wire was inserted.

## Results and Discussion

We found two simultaneous processes during these sintering experiments. The first one is more like a standard sintering process (figure 1) while the second one is a carbonization process (figure 2).

### Sintering

It has been shown that sintering of nanoparticles at the beginning is spontaneous and does not require applying additional heat and the process is only prevented by the stabilizing shell that surrounds the nanoparticles [14], [37]. This is comparable to others in-situ TEM experiments of cold welding of ultrathin gold nanowires, showing that no additional heat or pressure is required to weld gold nanowires together within seconds [38].

In figure 1, a coalescence process is shown. The starting structure (figure 1a) shrunk (figure 1b) after the first 31 s of increasing electrical power while the resistance decreased by about an order. We interpret this as that the shells around the nanoparticles must have been altered, possible due to carbonization from the Joule heating and a sintering process has therefore followed as the particles agglomerate and form a more homogeneous structure but do not completely melt together, as seen in the figure, until more Joule heating is applied.

This sintering step occurred in less than one second, this is not surprising as other coalescence studies of nanoparticles have shown that the main driving force is linked to the high surface area to volume ratio property of the nanoparticles and that the lowering of surface energy during coalescence allows for heat release which drives the coalescence [39], [40].

It has been shown that for very small particles, less than 5 nm, the coalescence speed is instantaneous and more like liquid drops merging [41]. Nanoparticles less than 10 nm in diameter will also benefit from rotation in which nanoparticles, can rotate and align themselves to each other during coalescence which in turn increases the speed of coalescence [37], [42].

Bigger particles, as in our case, merge through neck-formation by a surface diffusion process [42], [43]. The most limiting speed factor of this process is the substrate which can change the coalescence speed of the particles by several order of magnitude [41]. If the nanoparticles are bound strongly to the surface the process of coalescence is governed by an Ostwald ripening process, or if they are loosely attached by a faster Brownian motion process [42]. It is likely that in our experiment the altered shell around the nanoparticles will have similar effects in the beginning of the coalescence process as a substrate would have.

When the particles coalescence and start to form larger structures as in our experiment, the ability to melt and coalescence further is becoming more and more difficult due to increase of the melting temperature, conduction of heat to a larger mass and loss of heat from coalescing boundaries to the hole structure [42].

By increasing the Joule heating the process was enabled to continue and a grain growth phase started (figure 1c) and the grain sizes increased. The resistance in this phase was usually lower than for only sintered samples, going down to fractions of k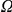. The power density was 0.15 mW/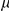
m^3^.

### Carbonization

The arrow in figure 1d points to a structure that becomes visible after the structure broke, which we interpreted as a residue of carbon. In figure 2 and the supplementary movie S1, this carbonization process is shown more clearly. Instead of particles melting and coalescence as in figure 1, the particles here evaporated away, leaving behind a net of carbon. This process was visible in about half of the samples, depending on the particular geometry of the structure.

**Figure 3:**
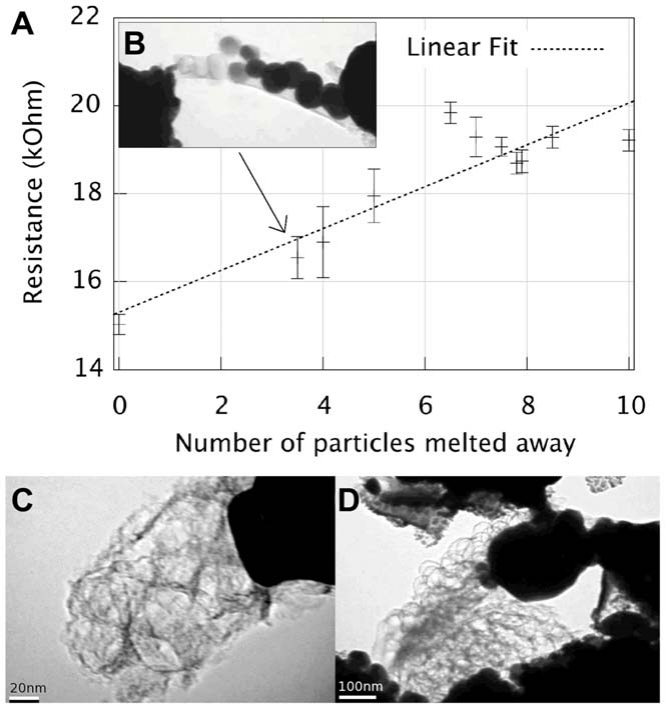
Electrical resistance as function of number of melted silver nano particles. a) Assuming a zero resistance of the silver particles, the initial measured resistance of 15 k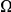 can be interpreted as the contact resistance. During heating, the resistance increases by a total of 5 k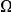 when all particles have been evaporated, this increase is interpreted as the total resistance of the net. The fractional numbers of particles are due to image exposure time, at some instances a gray-shaded particle is visible meaning that it evaporated away during this frame. This is taken into account as giving it a fractional count. b) shows an inset of a TEM image of the sample corresponding to the marked measurement point in the plot. c) Higher magnification of the structure shown in figure 1d. d) A larger cluster with a resistance of 6 k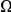.

The net-like structure left behind indicate that the carbon structure was stable during the evaporation of the nanoparticles. Figure 3c–d shows carbon nets when all silver particles were evaporated. The spherical shape of each carbon cage resembles quite well the particle that disappeared. This is particular visible in figure 2. Similar looking carbon remains have been seen by others in evaporation of gold nanoparticles on carbon nanotube substrate [44].

Furthermore the silver content in the carbon net should also be very low since the solubility of C in liquid silver near the melting point is less than 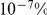 [45]. When we continued the Joule heating even further, at much higher power levels (of about 290 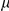
W), the carbon net abruptly evaporated (carbon melts at 3652°C).

Before the carbonization phase the resistance slowly decreased about an order of magnitude from the initial resistances that were in the M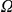 range, figure 2e. No visible change in the structure were observed in the TEM images in this phase, figure 2a.

During the carbonization the resistance dropped sharp by 1–2 orders of magnitude and at power levels between 1 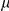
W and 100 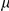
W, corresponding to power densities of about 0.1 to 10 mW/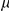
m^3^. To calculate these power densities, the volumes were estimated from the area measured in the TEM images and modeled as a square rod with its length set to the distance between the two probes.

The reason for this sharp decrease in resistance at the beginning could be due to several mechanisms including contact resistance curing and carbonization. The similarities between the resistance curves in figure 2e, however, point towards a common phenomena such as carbonization and not to curing of high ohmic contacts that should be more randomly distributed. Others have also reported on similar resistance curves due to electric heating of nano ink [46]. Another reason for the carbonization interpretation was the lack of continuous metallic connection between the nanoparticles as evident from the TEM images, like the gap marked by an arrow in figure 2a.

Carbonization is a complex process with many concurrently reactions such as dehydrogenation and isomerization. The final temperature applied, controls the degree of carbonization and the residual content of foreign elements, for example, at T∼900°C the carbon content of the residue exceeds a mass fraction of 90 wt. %, whereas at T∼1300°C more than 99 wt. % carbon is found [47]. In our case, we know the temperature when the silver particles melts (960°C) and at this stage the carbonization should be quite complete. Furthermore a previous thermogravimetric (TGA) study also states that the solvent can burn-off at a low temperature [8]. In air it has also been show that the temperature for decomposition of the solvent goes down to below 200°C via an exothermic process [13].

Others have shown that the sintering temperature of nanoparticles can be controlled by selecting organic-binders with different burn-off temperature characteristics as sintering starts directly after the binder has been burned-off [8].

In our experiments we observe a similar effect with the carbonization of the binder that in turn is the starting trigger for the sintering process. The heating rate was similar for all the experiments but in the necklace type of experiments the nanoparticles evaporated instead of sintering. The shape and size of the different samples is the major difference between the conducted experiments. Therefore it is more likely that this is the governing parameter if evaporation or sintering would occur after the carbonization has taken place rather than the degree of carbonization. The carbonization leads to a carbon-net structure that is hollow and in three of the experiments the silver metal was observed floating through the net structure. However when carbonization occurs electrical conductivity instantly increases and this results in an instant increase of electrical power input into the sample. A series resistor was connected to the system to balance this feedback effect, limiting it, but not removing the effect completely. If the sample has different geometry this will result in different amount of cooling (radiation and conduction) and for the necklace type of geometry this leads to evaporation of the nanoparticles.

By studying the change in resistance of the sample when the particles evaporated, one by one (figure 3a), and assuming a zero resistance of the silver particles, the initial measured resistance of 15 k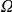 can be interpreted as the contact resistance, thus the metal particles short-circuit the net and most of the electrical current will therefore go through the particles. Even if the particles are not in contact with each other they still can interact with the net by donating electrons into it, i.e. short-circuit small portions of the net, and therefore after evaporation of the particles a resistance increase would occur [48], [49]. During heating, the resistance did increase by 460 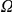 for each evaporated particle leading to a total increase in resistance of 5 k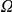 when all particles have been evaporated, this total resistance increase is interpreted as the total resistance of the net. If we model the carbon net as a simple tube with length and diameter corresponding to the net the conductivity is 4 10^5^ Sm^−1^, which is of the same order as graphite and graphitized carbon nanowires [50].

The reproducibility was high if one compares the similarities of the electrical resistance measurements curves from the experiments (figure 2e) and in all the necklace experiments the silver evaporated away but for the bigger clusters coalescence and sintering occurred more frequently.

The TEM electron beam does have an effect on organic compounds such as the steric stabilization shells around the silver nanoparticles in the ink. Others have shown that in an experiment with alkanethiol-passivated gold nanoparticles the effect of destroying the organic shell depends on beam energy as well as beam dose and in that case a beam energy level of 200 keV and at dose rates of tens of 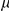
C/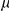
m^2^ were needed before any effect could be observed [51]. In our experiments we used a beam energy of 160 keV and beam dose rates of at most 0.5 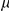
C/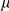
m^2^ so it is likely that beam-effects in our experiment should be minor.

### Relevance for printed nanoink heated in non-inert atmosphere (air)

We found little correlation of the sample size and the power required to sinter the nanoparticles together, which was not surprising considering the highly different geometry of the samples, however, the order of magnitude of power used can be compared with the ones reported in the earlier macroscopic studies. Our results are 3–4 orders larger than earlier reports [31], but should be consistent with their finding, considering that the volume used in their calculations are the total volume of a macroscopic silver film, while it is clear that only a filamentary low ohmic path is created in the electrical sintering process.

Evaporation of silver is not likely to happen within macroscopic inkjet samples at normal conditions. The reason for our observations is that the samples were very narrow with very few neighboring particles. Within a large sample the vapor from an evaporated particle will condense on neighboring particles and coalescence instead.

Because of the oxygen present in the atmosphere, burning of carbon should, on the other hand, be present in Joule heating of macroscopic samples in air. However, carbonization in air can not entirely be ruled out if one consider that the printed structure might not be homogeneously sintered. If the surface of the structure is sintered first, the interior parts might be protected from oxygen, and a similar carbonization process as found in this study might be present.

Others have shown that by embedding polymers into the ink will give a more durable structure [52]. The polymers will prevent microcracking of the sintered structure that otherwise occurs due to the burn-off of the solvent [26]. If it is possible to carbonize the solvent instead of completely burning it off will of course help in preventing microcracking of the sintered structure in a similar way as adding polymers.

Surface oxidation of nanoink during sintering in air is another problem thus the presence of oxides increases the required annealing temperature [22]. To prevent oxidation, sintering by intense pulsed light (i.e. camera flash) has recently been suggested [29]. Sintering in inert atmosphere can be another route and the carbonization process might then be usable in printed electronics applications, as carbon itself is a good conductor and is an environmentally friendly material suitable for large scale printed electronics. To explore whether an all carbon Joule heating process is possible would be interesting.

### Conclusion

In this study when the ink was Joule heated, the first cause of conductivity increase was the carbonization of the solvent. The electrical conductivity of the carbonized shells are of the same order as graphite. Because this happened at a rather low temperature, this is most important for a low-temperature demanding application such as printing on paper and if the shells are carbonized instead of burning off this might be useful in preventing microcracking as this limits shrinking effects.

Further, we found an onset of Joule heating induced carbonization and sintering at power levels of 0.1–10 mW/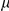
m^3^, either the coalescence take place right after the protective shells around the nanoparticles have been carbonized or the nanoparticles are completely evaporated. If coalescence or evaporation will take place depends on the particular geometry of the sample and if the particles coalescence, to continue the sintering process with grain growth an increased power input was necessary.

## Supporting Information

Movie S1 **Joule heating of nanoink by in-situ TEM-STM method.** The movie shows the carbonization and the sintering process followed by evaporation of the silver nanoparticles.
(MP4)
